# A review of the effects of incubation conditions on hatchling phenotypes in non-squamate reptiles

**DOI:** 10.1007/s00360-021-01415-4

**Published:** 2022-02-10

**Authors:** Christopher R. Gatto, Richard D. Reina

**Affiliations:** School of Biological Sciences, Monash University, 25 Rainforest Walk, Clayton, VIC 3800 Australia

**Keywords:** Egg-laying reptiles, Incubation environment, Hatchling, Phenotype, Development

## Abstract

Developing embryos of oviparous reptiles show substantial plasticity in their responses to environmental conditions during incubation, which can include altered sex ratios, morphology, locomotor performance and hatching success. While recent research and reviews have focused on temperature during incubation, emerging evidence suggests other environmental variables are also important in determining hatchling phenotypes. Understanding how the external environment influences development is important for species management and requires identifying how environmental variables exert their effects individually, and how they interact to affect developing embryos. To address this knowledge gap, we review the literature on phenotypic responses in oviparous non-squamate (i.e., turtles, crocodilians and tuataras) reptile hatchlings to temperature, moisture, oxygen concentration and salinity. We examine how these variables influence one another and consider how changes in each variable alters incubation conditions and thus, hatchling phenotypes. We explore how incubation conditions drive variation in hatchling phenotypes and influence adult populations. Finally, we highlight knowledge gaps and suggest future research directions.

**Supplementary Information:**

The online version contains supplementary material available at 10.1007/s00360-021-01415-4.

## Introduction

Reproductive life-history modes can broadly be described as ranging from oviparity with little or no parental care to viviparity with parental care, and a variety of intermediate forms (Lodé 2012). Modes generally differ in their degree of parental investment in individual offspring, with a trade-off between offspring number and probable survival rate (Blackburn 1999). Oviparity with little or no parental care reduces the time females spend burdened by eggs, both physically and physiologically (Blackburn 1999), but exposes eggs to variations in the incubation environment, including unfavourable conditions that may negatively affect embryonic development (Angilletta et al. 2000; Rana 1990). While many oviparous species have evolved adaptations (e.g., lecithotrophic viviparity or post-ovipositional brooding) to reduce environmental variation for developing eggs and embryos, most reptile species do not provide any parental care during or after incubation (Balshine 2012). Thus, the timing of oviposition and location of clutches can have important implications for incubation conditions and, therefore, the quality and quantity of resultant offspring (Ackerman 1991; Kamel and Mrosovsky 2004; Kolbe and Janzen 2002).

Research into how different incubation environments influence reptile hatchling phenotypes has been extensive (e.g., Ackerman 1991; Ashmore and Janzen 2003; Bell et al. 2013; Booth 2006, 2017; Deeming 2004; Deeming and Ferguson 1991b; Gutzke et al. 1987; Hutton 1987; Janzen and Paukstis 1991; Mitchell et al. 2018; Noble et al. 2018b; While et al. 2018) and the significance of variation in incubation environments is clear (Hamann et al. 2010; Nelson et al. 2004; Rees et al. 2016). However, the majority of this research has focussed on the effects of temperature on embryonic development and in particular, the phenomenon known as temperature-dependent sex determination (TSD), which occurs in all reptile taxa except snakes (Shine 2003). Temperature has been shown to influence population viability by affecting the primary sex ratios (PSR) of developing embryos (Fuentes et al. 2010; Hanson et al. 1998; Hawkes et al. 2007; Mitchell and Janzen 2010; Mrosovsky 1994) as well as hatchling traits, such as locomotor performance and morphology (Booth and Evans 2011; Cavallo et al. 2015; Wood et al. 2014).

In contrast, much less attention has been paid to the impacts of other environmental factors, such as moisture, oxygen concentration and salinity, on hatchling phenotypes. These environmental variables, like temperature, can vary significantly both temporally and spatially. Moisture concentrations vary with depth, air humidity, rainfall and with the tides, oxygen concentrations decrease from atmospheric levels as microbial activity and embryonic metabolic rates increase and salt concentrations vary with proximity to the ocean, rainfall and tidal washing. Natural variation in each of these three environmental variables can have significant consequences on embryonic development. For example, flooding was identified as the main cause of mortality (29% of all eggs) in black caiman (*Melanosuchus niger*) nests (Villamarín-Jurado and Suárez 2007) and higher microbial loads in olive ridley nests (*Lepidochelys olivacea*) reduced nest oxygen concentrations and decreased hatching success (Bézy et al. 2015). Additionally, environmental variables can interact, modulating each other’s influence on embryonic development. Environmental variables can influence each other directly (e.g., increased moisture decreases nest temperatures, Charruau 2012; Tezak et al. 2018) or an influence the response of embryos to other environmental variables (e.g., hypoxia reduces the thermal tolerance of embryos, Smith et al. 2015a). These interactions make understanding and predicting the nest and incubation environment more complex. Without this information, it is difficult to predict with any certainty (1) how hatchling phenotypes will respond to changes in complex environmental systems and (2) the potential consequences for adult populations (Díaz-Paniagua and Cuadrado 2003). Unfortunately, the current focus on thermal effects and relative dearth of studies on other environmental variables limits our understanding of the dynamics of nests, both currently and under climate change.

There is a clear need to investigate how environmental factors other than temperature influence hatchling phenotypes and how these effects may vary among oviparous reptile taxa. However, comparisons among reptile species are complicated by the diversity of reproductive strategies (oviparity to viviparity), sex determination mechanisms (genetic, environmental or a combination) and the level of parental care during incubation (e.g., brooding). Thus, including all reptiles and their responses to altered incubation environments is too large a task for a single review. Therefore, in this review, we focus on the effects of incubation environment on oviparous non-squamate reptiles (i.e., crocodilians, testudines and tuataras, hereafter ‘non-squamates’), because they are underrepresented in the literature and because non-squamate reptiles share similar reproductive characteristics, compared to the high diversity found in squamates. The role of temperature in determining hatchling phenotypes are reviewed extensively elsewhere (Booth 2006, 2017; Deeming and Ferguson 1991b; Navara 2018; Noble et al. 2018b; Warner 2011; Warner and Shine 2008; While et al. 2018), and thus are not a major theme of our review. We instead primarily focus on how moisture, oxygen concentration and salinity influence hatchling phenotypes and developmental success in non-squamate reptiles. We discuss how these environmental factors can interact to determine phenotypes and explore the impact that climatic variation potentially may have on hatchling recruitment and population viability. Finally, we recommend future research directions to address underrepresented biological topics or taxonomic groups. Hopefully, exploring the effects of incubation conditions other than temperature in a broader range of taxa will lead to meta-analyses, like that of Noble et al. (2018b), that improve our understanding of how the incubation environment influences embryonic development and hatchling phenotypes in reptiles.

## Effects of incubation conditions on hatchling phenotypes

Incubation conditions are largely dependent upon the type of environment in which eggs are deposited. Reptiles exhibit substantial variety in egg-laying preferences across taxa: shallow underground nests laid in soil at a depth of 30–155 mm (e.g., tuataras *Sphenodon punctatus*; Thompson et al. 1996); deep underground nests laid in sand at a depth of up to 76 cm (e.g., leatherback turtles *Dermochelys coriacea*; Billes and Fretey 2001); nests that flood (e.g., northern long-necked turtles *Chelodina rugosa*; Kennett et al. 1993)*;* and aboveground mounds built from vegetation (e.g., crocodilians; Webb and Manolis 1998). Each of these preferences has consequences for one or more environmental variables affecting the nest microenvironment. For example, shallow or aboveground nests are likely to experience greater fluctuations in temperature than those laid deep underground (Booth 2006), and deeper nests typically being warmer than average ambient air temperatures due to metabolic heating (Seymour and Ackerman 1980; Sieg et al. 2011). In this section, we review how developing embryos are affected by variations in environmental factors during incubation. However, effects of geographically large-scale and long-term climatic variation are beyond the scope of the initial review in this section. We briefly discuss the potential consequences of large-scale temporal and spatial variation in Sects. 3 and 4. In this section, we focus on the effects of abiotic factors that alter incubation conditions and influence developing embryos. Thus, the effect of biotic factors and parental care on incubation conditions, offspring phenotypes and offspring survival are also beyond the scope of this section of the review.

### Temperature

#### Summary

The role of temperature during incubation in determining hatchling traits has been extensively studied and reviewed (Booth 2006; Deeming 2004; Janzen and Paukstis 1991; Noble et al. 2018b; While et al. 2018; Wibbels 2003) and temperature is generally considered the most influential environmental variable for developing embryos. In this section, we provide a brief summary of temperature’s effects on hatchling phenotypes to provide context for the overall impact of temperature on embryonic development compared to other environmental variables.

#### Sex ratio

Temperature has been the most studied environmental factor influencing hatchling phenotypes in reptiles. In particular, research has focused on the effect of temperature on sex determination (i.e. TSD), and there are a number of detailed reviews on reptile sex ratio responses and likely mechanisms (Castelli et al. 2020; Janzen and Paukstis 1991; Lang and Andrews 1994; Merchant-Larios and Diaz-Hernandez 2013; Rhen and Schroeder 2010; Schwanz and Georges 2021; Valenzuela and Lance 2004; Wibbels 2003). Despite the plethora of studies investigating TSD in reptiles, knowledge of the mechanisms of TSD remains elusive.

There are three main patterns in the response of sex to temperature. FMF (female–male–female) is a pattern in which males are observed at intermediate temperatures and females at higher and lower temperatures. FM (female–male) and MF (male–female) patterns only transition between the sexes once, with FM species producing females at lower temperatures and MF species producing females at higher temperatures.

TSD occurs during the temperature-sensitive period, which is generally the middle third of development in most reptiles (Girondot et al. 2018). Pivotal temperatures—the range at which a clutch produces 50% males and 50% females—remain relatively consistent within species apart from small variations between geographically distinct sub-populations (Ewert et al. 2005). In contrast, pivotal temperatures vary significantly within the Testudines and among taxonomic Orders (Table S1).

#### Body size

Morphological variation (e.g., length, width, mass) in response to incubation temperature varies significantly among non-squamates (Table S2). For example, in the Testudines, turtle bodies are typically longer and wider at lower incubation temperatures, but generally do not vary in mass (Booth and Astill 2001; de Souza and Vogt 1994; Gutzke and Packard 1987; Micheli‐Campbell et al. 2011). In contrast, crocodilian hatchling length and mass generally display no response to incubation temperature (Allsteadt and Lang 1995; Hutton 1987; Joanen et al. 1987).

It has been suggested that temperature affects morphology by altering biochemical reactions and the resultant rate of embryonic development (Booth 2017). Increasing temperature may increase reaction rates until they disrupt protein structures, modify protein and gene expression or a combination of both (Singh et al. 2020). Short periods of extreme temperatures generally produce shorter and lighter hatchlings in sea turtles (Maulany et al. 2012; Sim et al. 2015).

#### Locomotor performance

The effect of temperature during incubation on locomotor performance has been extensively examined in sea turtles (see review by Booth 2017), but much less so in other reptile taxa. Optimal incubation temperatures vary among and within taxa (Table S3) but extended or repeated periods of high temperature during incubation consistently have negative effects on hatchling locomotor performance in sea turtle species (Maulany et al. 2012; Sim et al. 2015). Incubation conditions can influence locomotor performance directly via embryonic muscle development and indirectly via effects on body size (Booth 2017).

#### Hatching success and developmental rate

Non-squamates vary significantly in their response to chronic and acute heat stress (Hall and Sun 2020), although hatching success rates in non-squamates are generally highest at intermediate temperatures (Table S4), with embryonic death occurring at extreme high or low temperatures.

Developing embryos appear quite resilient to short-term extreme temperatures, although the cumulative length of exposure has the largest effect on embryonic mortality in sea turtles (Bladow and Milton 2019; Howard et al. 2014; Lang and Andrews 1994; Maulany et al. 2012; Sim et al. 2015). The temperature at which mortality occurs can differ by up to 12 °C depending on whether embryos of the same species experience either acute or chronic changes in temperature (Taylor et al. 2020).

### Respiratory gases

#### Summary

Diffusion is the driver of oxygen into non-squamate eggs. In clutches laid above ground, oxygen quickly diffuses into the egg, while in underground nests, oxygen must first diffuse through the substrate along a concentration gradient (Hillel 2003; Prange and Ackerman 1974). As a result, oxygen concentrations within underground nests are influenced by a number of physical factors (e.g., depth, moisture content, temperature) and characteristics of the nest substrate (e.g., grain size, rugosity, pore spacing) (Ackerman 1980; Lutz and Dunbar-Cooper 1984). Similarly, oxygen availability can be reduced due to surrounding biotic influences (e.g., proximity to other nests, clutch size, microbes or organic material) and increased metabolic demands of embryos at later stages of development (Ackerman 1980; Bézy et al. 2015; Lutz and Dunbar-Cooper 1984). As developing embryos consume oxygen throughout incubation, they produce carbon dioxide resulting in increasing carbon dioxide concentrations within the nest (Booth 1998, 2000; Booth and Dunstan 2018; Garrett et al. 2010; Lutz and Dunbar-Cooper 1984). Thus, studies that reproduce natural nest conditions should consider not only oxygen concentrations but also carbon dioxide concentrations. The diffusion of oxygen into eggs depends on each species’ eggshell structure. Rigid crocodilian eggs are approximately five times less permeable to oxygen compared to parchment-like sea turtle eggs (Ackerman et al. 1985a; Ackerman and Prange 1972; Lutz et al. 1980).

Some non-squamate species such as turtles and tuataras are able to arrest embryonic development in response to reduced oxygen levels, although the stage of development at which development can successfully be arrested varies (Rafferty and Reina 2012). In freshwater and sea turtles, low oxygen levels (~ 1%) within the oviducts allow females to arrest the embryonic development of eggs until oviposition (Rafferty et al. 2013; Williamson et al. 2017b). However, once embryonic development has commenced, embryos require a relatively constant supply of oxygen and cannot re-arrest if exposed to hypoxic conditions (Williamson et al. 2017b). Unlike sea turtle embryos, crocodilians do not appear capable of extending embryonic arrest post-oviposition (Williamson et al. 2017a), while tuataras have only been shown to be capable of arresting embryonic development during gastrulation (Moffat 1985; Rafferty and Reina 2012). Further studies are required in other taxa.

#### Sex ratio

Research on the effect of oxygen concentration on sex determination in non-squamates is limited. Studies in the Testudines (Etchberger et al. 1991) and crocodilians (Deeming and Ferguson 1991a) have found no relationship between oxygen concentration during incubation and sex determination (Table 1). Further research is required to discover if the same is true in tuataras.

**Table 1:** The effect of incubation oxygen, carbon dioxide, moisture and salt concentrations on mass, morphology, post-hatching growth rates and sex determination

	With hyperoxic conditions	With hypoxic conditions	With normoxic conditions	No effect of oxygen concentrations
Turtle	Heavier: Liang et al. (2015)			Mass: Etchberger et al. (1991)
	Larger body size: Rings et al. (2014)^A^ Liang et al. (2015)		Smaller body size: Rings et al. (2014)^A^	
	More females in hypercapnic conditions: Etchberger et al. (2002)			Sex determination: Etchberger et al. (1991)
			Higher survival rate: 2 months; Liang et al. (2015)^B^	Survival: 2 months; Liang et al. (2015)^B^
				No effect of hypercapnia on sex determination: Etchberger et al. (1992)
				No effect of hypercapnia on survival: 45 days; Etchberger et al. (1992)
Crocodile		Lighter: Warburton et al. (1995)		
		Smaller body size: Warburton et al. (1995)		

	With higher moisture concentrations	With drier moisture concentrations	With intermediate moisture concentrations	No effect of moisture concentrations
Turtle	Heavier: Bobyn and Brooks (1994) Brooks et al. (1991) Cagle et al. (1993) Finkler (1999) Hewavisenthi et al. (2001) Janzen (1993) Miller et al. (1987) Miller (1993) Packard et al. (1987) Packard et al. (1988) Booth (2002) Miller and Packard (1992) Packard and Packard (1986) Packard and Packard (1989) Packard et al. (1981) Packard et al. (1982a) Packard et al. (1985a) Tracy et al. (1978)	Heavier: Bodensteiner et al. (2015)^C^		Mass: Erb et al. (2018) Gatto and Reina (2020) Gatto et al. (2021) Reece et al. (2002) Ratterman and Ackerman (1989) Bodensteiner et al. (2015)^D^
	Larger body size: Cagle et al. (1993) Gutzke et al. (1987) Hewavisenthi et al. (2001) Janzen (1993) Miller et al. (1987) Miller (1993) Packard et al. (1989b) Tezak et al. (2020) Booth (2002) Miller and Packard (1992) Packard et al. (1981) Packard et al. (1982a) Packard et al. (1985a) Bodensteiner et al. (2015)^D^	Larger body size: McGehee (1990) Bodensteiner et al. (2015)^C^		Body size: Erb et al. (2018) Gatto and Reina (2020) Gatto et al. (2021) Reece et al. (2002)
	More males: Lolavar and Wyneken (2021) LeBlanc and Wibbels (2009) Paukstis et al. (1984)			Sex determination: Bobyn and Brooks (1994) Brooks et al. (1991) Packard et al. (1987) Packard et al. (1989b) Packard et al. (1985a) Lolavar and Wyneken (2017)^E^ Bodensteiner et al. (2015)^C^
	Faster growth rates: Erb et al. (2018)			Hatchling growth rates: 23 months; Bobyn and Brooks (1994) 7 months; Brooks et al. (1991) 14 weeks; McKnight and Gutzke (1993) 50 days; Miller (1993)
				Survival: 23 months; Bobyn and Brooks (1994) Duration not reported; Janzen (1993)
				Abnormalities: Gutzke et al. (1987) Hewavisenthi et al. (2001)
Tortoise				Mass: Spotila et al. (1994)
Tuatara				Mass: Thompson (1990)
				Body size: Thompson (1990)

	With higher salt concentrations	With lower salt concentrations	With intermediate salt concentrations	No effect of salt concentrations
Turtle		Heavier: Bower et al. (2013)		
		Longer: Bower et al. (2013)		

Studies are allocated based on the conditions that produced the largest hatchlings and fastest growth rates. For sex determination, we only include studies that directly determined sex and not those that estimated sex using temperature

^A^Gas treatments maintained for 5 days post-oviposition then all eggs exposed to normoxia (21% O_2_)

^B^Higher survival with normoxic conditions during incubation when incubation occurred at 34 °C but there was no effect of oxygen concentration when incubation temperature was 26.5 °C

^C^In situ nests in 2013

^D^In situ nests in 2012

^E^Manipulated the amount of evaporation by controlling relative humidity (range: 76–94.8% RH) and maintained sand moisture by spraying with water at either 29 °C or 25 °C

#### Body size

Higher concentrations of oxygen during incubation generally result in larger hatchlings in all non-squamate taxa (Table 1), while lower oxygen concentrations likely limit the metabolism of embryos, resulting in reduced conversion of yolk into hatchling mass (Ackerman 1981; Etchberger et al. 1991; Liang et al. 2015; Warburton et al. 1995).

#### Locomotor performance

The effect of oxygen concentration during incubation on locomotor performance is complex and varies among non-squamates (Table 2). Chinese soft-shelled turtles (*Pelodiscus sinensis*) maintained at 22% oxygen for the entirety of incubation were faster crawlers compared to hatchlings incubated at 12% or 30% oxygen (Liang et al. 2015). However, this effect was only observed at very high incubation temperatures of 34 °C, but not at 26.5 °C. The effect of oxygen concentration may have been greater at 34 °C than at 26.5 °C because of the increased metabolic demands of embryos at higher temperatures. Additionally, hatchlings incubated in hyperoxia did not exhibit improved locomotor performance compared to those incubated at normoxia. In contrast, flatback sea turtle (*Natator depressus*) hatchlings incubated in hyperoxic air (42% oxygen) for the first 5 days followed by normoxia for the remainder of incubation were faster crawlers but slower swimmers than hatchlings incubated entirely at normoxia (21% oxygen), though the long-term fitness advantages of hyperoxia remain unclear (Rings et al. 2014).

**Table 2:** The response of various measures of locomotor performance to different incubation environments

Locomotor trait	Environmental variable	Response	Range	Species	References
Time to self-right	Moisture	No effect	4 = 6 = 8 (% w/w)	*Chelonia mydas*	Gatto and Reina (2020)
		Faster at higher moistures	4 > 6 = 8 (% w/w)	*Natator depressus*	Gatto and Reina (2020)
			4 > 6 > 8 (% w/w)	*Lepidochelys olivacea*	Gatto and Reina (2020)
	O_2_	No effect	0 = 21 = 42 (% O_2_)	*Natator depressus*	Rings et al. (2014)^A^
Successful self-righting attempts	Moisture	No effect	4 = 6 = 8 (% w/w)	*Chelonia mydas*	Gatto and Reina (2020)
		More successful attempts at higher moistures	4 < 6 < 8 (% w/w)	*Natator depressus*	Gatto and Reina (2020)
			4 < 6 < 8 (% w/w)	*Lepidochelys olivacea*	Gatto and Reina (2020)
	O_2_	No effect	0 = 21 = 42 (% O_2_)	*Natator depressus*	Rings et al. (2014)^A^
Crawling speed	Moisture	No effect	4 = 6 = 8 (% w/w)	*Chelonia mydas*	Gatto and Reina (2020)
			4 = 6 = 8 (% w/w)	*Natator depressus*	Gatto and Reina (2020)
			− 150 = − 950 (kPa)	*Chelydra serpentina*	Janzen (1993)
		Faster at higher moistures	4 < 6 < 8 (% w/w)	*Lepidochelys olivacea*	Gatto and Reina (2020)
			− 150 > − 850 (kPa) 111 > 18 (% w/w)	*Chelydra serpentina*	Miller et al. (1987)
			− 150 > − 850 (kPa) 111 > 18 (% w/w)	*Chelydra serpentina*	Finkler (1999)
	O_2_	No effect	12 = 22 = 30 (% O_2_)	*Pelodiscus sinensis*	Liang et al. (2015)^B^
		Faster at higher O_2_	0 = 21 < 42 = 0 (% O_2_)	*Natator depressus*	Rings et al. (2014)^A^
		Faster in normoxia	22 > 12 = 30 (% O_2_)	*Pelodiscus sinensis*	Liang et al. (2015)^B^
Swimming speed	Moisture	Faster at higher moistures	− 150 > − 850 (kPa) 111 > 18 (% w/w)	*Chelydra serpentina*	Miller et al. (1987)
			− 150 > − 850 (kPa) 53 > 10 (% w/w)	*Chelydra serpentina*	Miller (1993)
	O_2_	Faster at lower O_2_	0 = 21 > 42 (% O_2_)	*Natator depressus*	Rings et al. (2014)^A^
Mean swim thrust	Moisture	No effect	4 = 6 = 8 (% w/w)	*Chelonia mydas*	Gatto and Reina (2020)
			4 = 6 = 8 (% w/w)	*Natator depressus*	Gatto and Reina (2020)
			4 = 6 = 8 (% w/w)	*Lepidochelys olivacea*	Gatto and Reina (2020)
Time spent powerstroking	Moisture	No effect	4 = 6 = 8 (% w/w)	*Chelonia mydas*	Gatto and Reina (2020)
			4 = 6 = 8 (% w/w)	*Natator depressus*	Gatto and Reina (2020)
			4 = 6 = 8 (% w/w)	*Lepidochelys olivacea*	Gatto and Reina (2020)
Thermal tolerance	Moisture	No effect	5 = 8 (% v/v)	*Chelonia mydas*	Gatto et al. (2021)

The oxygen, carbon dioxide, moisture and salt concentration at which each trait is highest and lowest is identified

^A^Gas treatments maintained for 5 days post-oviposition then all eggs exposed to normoxia (21% O_2_)

^B^Effect on crawling speed occurred when incubation temperature was 34 °C, but there was no effect when incubation temperature was 26.5 °C

#### Hatching success and developmental rate

The absolute oxygen consumption of embryonic non-squamates increases as embryos develop and grow larger. In the Nile soft-shelled turtle (*Trionyx triunguis*), peak oxygen consumption occurred at ~ 82% of incubation, before decreasing by ~ 30% for the remainder of incubation (Leshem et al. 1991). Similar patterns have been observed in other turtles (Booth and Astill 2001) and crocodilians (Thompson 1989; Whitehead and Seymour 1990). Unlike testudines and crocodilians, the oxygen consumption of embryonic tuataras does not decrease before hatching (Thompson 1989). Thus, non-squamate embryos generally become more susceptible to hypoxia-induced mortality as they develop (Booth 2000; Cedillo-Leal et al. 2017; Cordero et al. 2017). Even a few hours of hypoxia can reduce hatching success (Ackerman 1981; Pike et al. 2015), as can maintaining embryos in hypoxia-induced arrest for extended periods (Table 3; Rafferty et al. 2013). Hypoxia, when combined with hypercapnia (see below), can decrease embryonic growth rates (Booth et al. 2020). Low oxygen and high carbon dioxide concentrations occur regularly in nests (Booth et al. 2020; Lutz and Dunbar-Cooper 1984) and thus, it is likely that gas concentrations regularly slow development rates. It is unlikely that developing embryos experience hyperoxia (i.e. atmospheric oxygen tensions above 21%) under natural conditions. However, studies have shown that hyperoxia does not generally result in higher hatching success compared to normoxia (Etchberger et al. 1991; Liang et al. 2015; Rings et al. 2014)*.* Therefore, increasing oxygen concentration above 21% does not appear to be a viable way of increasing hatching success in species with high embryonic mortality, such as leatherback sea turtles. Oxygen concentrations in the centre of underground nests are generally lower than those on the edges (Wallace et al. 2004), resulting in reduced hatching success in eggs in the centre of the nest compared to the periphery (Ralph et al. 2005). Therefore, increasing oxygen concentrations from low levels (e.g., 12–16 kPa in sea turtle nests Ackerman et al. 1997; Chen et al. 2010) regularly found at the end of incubation to atmospheric levels may improve hatching success.

**Table 3:** Minimum and maximum hatching success in various non-squamate taxa and the oxygen, carbon dioxide, moisture and salt concentrations that produced those results

Environmental variable	Order	Family	Species	Maximum hatching success	Concentration	Minimum hatching success	Concentration	Substrate	References
O_2_	Testudines	Cheloniidae	*Caretta caretta*	Max recorded: 86%	Max recorded: 20.1% O_2_	Min recorded: 0%	Min recorded: 16.2% O_2_	Sand	Bézy et al. (2015)^A^
			*Chelonia mydas*	94%	Early− stage: 19.79 kPa Middle− stage: 17.76 kPa Late− stage: 15.19 kPa	No effect of oxygen concentration		Sand	Chen et al. (2010)^B^
			*Natator depressus*	80% 85%	21% O_2_ 42% O_2_	15%	0% O_2_	No substrate then sand	Rings et al. (2014)^C^
		Dermochelyidae	*Dermochelys coriacea*	Range: 13–100%	Range: 17.1–19.9 kPa	No effect of oxygen concentration		Sand	Wallace et al. (2004)
			*Dermochelys coriacea*	~ 70%	~ 14.25 kPa	~ 20%	~ 19.5 kPa	Sand	Garrett et al. (2010)
		Emydidae	*Trachemys scripta*	11%	8% O_2_	77.1%	15% O_2_	Vermiculite	Etchberger et al. (1991)
		Trionychidae	*Pelodiscus sinensis*	73.7% 70%	22% O_2_ 30% O_2_	25%	12% O_2_	Vermiculite	Liang et al. (2015)^D^
				92–96%	12%, 22% & 30% O_2_	No difference among treatments			
O_2_ & CO_2_ CO_2_	Testudines	Emydidae	*Trachemys scripta*	96%	0% CO_2_, 21% O_2_	0%	15% CO_2_, 10% O_2_	Vermiculite	Etchberger et al. (2002)
		Cheloniidae	*Caretta caretta*	99–100%	21% O_2_, 0% CO_2_ 17% O_2_, 4% CO_2_ 14% O_2_, 7% CO_2_ 10% O_2_, 11% CO_2_	No difference among treatments		Sand Sand	Booth et al. (2020)^E^
			*Chelonia mydas*	97–100%	21% O_2_, 0% CO_2_ 17% O_2_, 4% CO_2_ 10% O_2_, 11% CO_2_				
	Testudines	Dermochelyidae	*Dermochelys coriacea*	~ 70%	~ 5.75 kPa	~ 10%	~ 2 kPa	Sand	Garrett et al. (2010)
		Emydidae	*Graptemys pseudogeographica kohnii*	85.7%	0% CO_2_	50%	10% CO_2_	Vermiculite	Etchberger et al. (2002)
			*Trachemys scripta*	96.7% 100% 93.3% 86.7%	0% CO_2_ 5% CO_2_ 10% CO_2_ 15% CO_2_	No difference among treatments		Vermiculite	Etchberger et al. (1992)
Moisture	Testudines	Chelidae	*Chelodina expansa*	100%	− 100 kPa − 350 kPa − 750 kPa	No differences among treatments		Vermiculite	Booth (2002)
		Cheloniidae	*Caretta caretta*	85.6% 70%	6% w/w 12% w/w	32% 19%	18% w/w 24% w/w	Sand	McGehee (1990)
				~ 86–91%	2.5–5.9% w/w	No differences among treatments		Sand	Tezak et al. (2020)
				0–86%	2.4–6.2% w/w	No effect of moisture concentration		Sand	Bézy et al. (2015)
				~ 90%	~ 5% v/v	~ 50% ~ 45%	5% v/v 7% v/v	Sand	Lolavar and Wyneken (2021)
				90.2% 90.2% 85.3%	10% v/v (94.8% RH) 6% v/v (76.5% RH) 8% v/v (76% RH)	No difference among treatments		Sand	Lolavar and Wyneken (2017)^F^
				84% 85% 86%	2–3% v/v 6–8% v/v 12–14% v/v	No difference among treatments		Sand	Lolavar and Wyneken (2020)
			*Chelonia mydas*	91–93.5%	4% w/w 6% w/w 8% w/w	No difference among treatments		Sand	Gatto and Reina (2020)
			*Lepidochelys olivacea*	63.3–71.7%	4% w/w 6% w/w 8% w/w	No difference among treatments		Sand	Gatto and Reina (2020)
			*Natator depressus*	76.7% 86.7%	6% w/w 8% w/w	43.3%	4% w/w	Sand	Gatto and Reina (2020)
				90%	200% w/w (− 180 kPa)	40–60%	10% w/w (− 3500 kPa)	Vermiculite	Hewavisenthi et al. (2001)
		Chelydridae	*Chelydra serpentina*	Not reported	172% w/w (− 150 kPa)	Not reported^C^	44% w/w (− 300 kPa) 22% w/w (− 800 kPa)	Vermiculite	Bobyn and Brooks (1994)^G^
				Not reported	171% w/w (− 100 kPa) 29% w/w (− 500 kPa)	No difference among treatments		Vermiculite	Brooks et al. (1991)
				75.3% 77%	− 150 kPa − 950 kPa	No difference among treatments		Vermiculite	Janzen (1993)
				87.5%	111% w/w (− 150 kPa)	68.8%	18% w/w (− 850 kPa)	Vermiculite	Miller et al. (1987)
				87%	1% w/w (− 150 kPa)	63%	0.6% w/w (− 950 kPa)	Sand	Packard et al. (1987)
				83%	113% w/w (− 150 kPa)	48%	17% w/w (− 950 kPa)	Vermiculite	Packard et al. (1987)
				67% 68%	53% w/w (− 150 kPa) 10% w/w (− 850 kPa)	No difference among treatments		Vermiculite	Miller and Packard (1992)
		Dermochelyidae	*Dermochelys coriacea*	Range: 0–5%	5% w/w 12% w/w	No effect of moisture concentration, very low hatching success		Sand	Bilinski et al. (2001)
		Emydidae	*Chrysemys picta*	94%	− 150 kPa	63%	− 1100 kPa	Vermiculite	Packard et al. (1989b)
				76% 76%	− 150 kPa − 650 kPa	48% 40%	− 1100 kPa − 1500 kPa	Vermiculite	Paukstis et al. (1984)^H^
				68%	− 150 kPa	0%	− 1500 kPa		
				100%	− 130 kPa	81.8% 83.3%	− 375 kPa − 610 kPa	Vermiculite	Packard et al. (1981)
				52% 54%	11% v/v 24% v/v	No difference among treatments		Soil	Bodensteiner et al. (2015)^I^
				82% 54%	17% v/v 32% v/v				
		Testudinidae	*Gopherus agassizii*	70%	0.4% w/w (− 5000 kPa)	16.5%	4% w/w (− 5 kPa)	Sand	Spotila et al. (1994)
	Rhynchocephalia	Sphenodontidae	*Sphenodon punctatus*	54.4% 68% 65.8%	− 90 kPa − 230 kPa − 400 kPa	No differences among treatments		Vermiculite	Thompson (1990)^J^
Salinity	Testudines	Chelidae	*Chelodina expansa*	94.4%	0 ‰	54.5%	70 ‰	Vermiculite	Bower et al. (2013)
		Cheloniidae	*Chelonia mydas*	53%	0% seawater (0 mg Cl^−^/ kg)	0%	75% seawater (1095 mg Cl^−^/kg) 100% seawater (1461 mg Cl^−^/kg)	Sand	Bustard and Greenham (1968)
		Chelydridae	*Chelydra serpentina*	~ 90% 100% ~ 87% ~ 92%	− 7 kPa (0 mOsm) − 188 kPa (77 mOsm) − 290 kPa (123 mOsm) − 542 kPa (235 mOsm)	~ 2%	− 2060 kPa (914 mOsm)	Sand	Rimkus et al. (2002)

^A^During the first half of incubation

^B^Mean value for in situ nests

^C^Gas treatments maintained for 5 days post-oviposition then all eggs exposed to normoxia (21% O_2_)

^D^Incubation temperature was 34 °C (top) and 26.5 °C (bottom)

^E^For 5.5 days, from 36 h to 7 days post-emergence

^F^Moisture concentrations maintained by spraying water at 29 °C for the 10% and 6% v/v treatments, and at 25 °C for the 8% v/v treatment

^G^Only reported hatching success of individual clutches and temperature treatments, did not report pooled results of moisture treatments

^H^Incubation temperatures were 4 h at 18 °C and 31 °C, respectively, with 8 h of gradual transition between the two temperatures (top) and 10 h at 19 °C and 26 °C, respectively, with 2 h of gradual transition between the two temperatures (bottom)

^I^Treatments applied in situ in 2012 (top) and 2013 (bottom)

^J^Only included data from 1986/87 season, because hatching success was very low in 1985/86

#### The role of carbon dioxide

Of the factors that limit oxygen supply to developing embryos, it is also important to consider the removal of carbon dioxide. Generally, factors that limit oxygen entry also limit carbon dioxide removal, which in buried nests leads to reduced oxygen concentrations near the centre of egg clutches (Ralph et al. 2005) and increased carbon dioxide levels (Ackerman et al. 1997). Studies that control oxygen concentration while manipulating carbon dioxide concentrations are limited, although laboratory research on freshwater and sea turtles have shown that higher carbon dioxide levels (10–15%) result in female-biased sex ratios (Table 1), slower development rates, longer incubation durations and smaller hatchlings with larger residual yolks compared to low carbon dioxide levels (0–5%) (Booth et al. 2020; Etchberger et al. 2002, 1992). In natural nests, embryonic carbon dioxide production (Booth 2000) and concentrations around the eggs increase throughout incubation (Lutz and Dunbar-Cooper 1984). Broad-shelled river turtle (*Chelodina expansa*) embryos are able to tolerate periods of hypercapnia (~ 6.7 kPa) for several successive days (Booth 1998), and hatching success in leatherback sea turtles was unaffected by maximum carbon dioxide concentrations of ~ 6 kPa carbon dioxide (Garrett et al. 2010). Carbon dioxide levels in natural nests vary, although periods of rain result in elevated carbon dioxide levels and carbon dioxide levels increase during incubation (Booth 1998). Species that lay their eggs above ground are less likely to experience elevated carbon dioxide levels, because the diffusion of gases is not impeded by substrate.

The effect of respiratory gases on embryonic development and hatchling phenotypes has been relatively unstudied in comparison to the effects of temperature and moisture but oxygen concentration has important implications for successful embryonic development, hatchling size and locomotor performance. It also appears to have strong interactions with both temperature and moisture that require further investigation. Carbon dioxide potentially plays a role in determining hatchling phenotypes, most notably hatchling sex as well as hatching success (Booth 1998; Etchberger et al. 2002, 1992). However, studies are restricted to the testudines and further investigation is required in other taxa. In comparison to carbon dioxide, oxygen concentrations have not been shown to influence sex determination, suggesting that carbon dioxide may directly influence hatchling phenotypes rather than indirectly by limiting oxygen availability to developing embryos. Studies on the effect of carbon dioxide on other phenotypes such as locomotor performance are limited. More studies on the role of oxygen and carbon dioxide during incubation are required, particularly in crocodilians.

### Moisture

#### Summary

Water exchange in reptile eggs is determined by a number of factors including the structure of the eggshell, the water potential and temperature of the nest and surrounding substrate and the percentage of the eggshell in contact with the surrounding substrate (Booth and Yu 2009; Packard 1999; Tracy et al. 1978). Reptilian eggshell structure differs among species and can be classified by shell thickness, flexibility and the presence or absence of a calcareous layer. Thicker, less flexible eggshells with a calcareous layer, as seen in crocodilians, are more resistant to the movement of water (Packard et al. 1982b). In comparison, chelonian eggshells generally have shells that are thinner, more flexible and despite also having a calcareous layers, allow greater movement of water (Packard et al. 1982b). Squamate eggshells are generally the thinnest and most flexible, with no calcareous layer (Packard et al. 1982b). Water moves across the eggshell, in liquid form or as water vapour, from high to low water potential (Thompson 1987). This movement of water may result the egg swelling with water or losing water to the surrounding environment. Water exchange, driven by differences in water potential, is generally greatest for eggs in direct contact with the surrounding substrate (Thompson 1987).

Egg size (specifically surface area to volume ratio) and egg water content can influence the response of developing embryos to moisture concentrations (Ackerman et al. 1985b; Gutzke and Packard 1985; Packard 1999). Large eggs exchange water more slowly than small eggs (Ackerman et al. 1985b), although under normal nest conditions females generally allocate enough water to eggs for successful development via the albumen (Ackerman et al. 1985b; Packard 1999; Packard et al. 1979). Within nests, eggs with a larger proportion of their surface in contact with the substrate generally absorb more moisture and produce larger hatchlings than eggs in the nest with more of their surface exposed to the air (Packard 1999; Packard et al. 1980; Tracy et al. 1978). Like larger eggs, larger clutches are less sensitive to changes in moisture content in the surrounding substrate compared to smaller clutches (Ackerman et al. 1985b).

#### Sex ratio

Nest substrate moisture and humidity levels during incubation may account for some of the observed variation in hatchling primary sex ratio (PSR) in species with TSD. Moisture indirectly alters nest temperatures (Lolavar and Wyneken 2015; Sifuentes-Romero et al. 2017b) and restricts oxygen availability (Cedillo-Leal et al. 2017), with potentially other direct, unknown mechanisms (Lolavar and Wyneken 2017). Potentially, moisture concentrations may alter the width of the transitional range of temperatures, as well as the upper and lower limits (Lolavar and Wyneken 2020). Moisture does not appear to modulate the pivotal temperature (Lolavar and Wyneken 2020).

Studies of both freshwater turtles (Gutzke and Paukstis 1983; LeBlanc and Wibbels 2009; Sifuentes-Romero et al. 2017a) and sea turtles (Lolavar and Wyneken 2015, 2017; Wyneken and Lolavar 2015) have shown that increased moisture during incubation results in increased production of male hatchlings (Table 1). However, other studies have found that moisture played no role in determining PSR in certain testudines (Bobyn and Brooks 1994; Hewavisenthi and Parmenter 2000; Packard et al. 1991) and one study in painted turtle hatchlings (*Chrysemys picta*) found that drier substrates produced more males than clutches incubated in wetter substrates (Paukstis et al. 1984).

However, it is difficult to compare these findings, because both substrate and arrangement of the eggs differs among studies. Experiments that use vermiculite or no substrate at all, and either partially cover or separate the eggs, do not represent natural nesting conditions. This can alter evaporative rates and moisture dynamics around the eggs, potentially influencing the response of the developing embryos to moisture. However, different substrates do not always alter the responses of embryos to incubation conditions (Packard et al. 1988). Additionally, studies differ in their measures of moisture, with some reporting water potential (kPa), while others report water concentration (%), which can be measured as weight/weight (w/w) or volume/volume (v/v). These inconsistencies make quantitative comparisons among studies investigating the effects of moisture on any reproductive or physiological trait difficult. Thus, in this review, we focus on identifying trends in the responses of embryos and hatchlings to moisture concentrations during incubation. Future meta-analyses will require standardised measures of hydric conditions that facilitate cross-study comparisons. Investigators should aim to measure water potential where possible, because water potential can be used to compare the amount of water available to embryos within different substrates and in different studies. In comparison, moisture concentration measured gravimetrically or volumetrically, cannot be compared among different substrates because of differences in substrate mass and grain size.

Furthermore, temperature and moisture strongly interact (Hill et al. 2015), making it difficult to isolate their individual effects on sex determination. Lolavar and Wyneken (2017) attempted to do this with sea turtle embryos by controlling evaporative cooling rates and maintaining all treatments at the same temperature. They found that nests subjected to evaporative cooling produced more males than nests that minimised evaporative cooling. Interestingly, all of the high moisture treatments in this study produced fewer females, irrespective of evaporative cooling rates, than would be expected based on temperature alone. A potential cause of this difference is that surface and internal egg temperatures are similar, but the difference between egg and air temperatures can be as high as 2 °C in sea turtle nests depending on humidity (Tezak et al. 2018). Thus, incubator air temperature measured in Lolavar and Wyneken (2017) may have been higher than the internal egg temperature, resulting in higher than expected male hatchling production. Discrepancies between incubator or air temperatures and egg temperature are potentially important sources of error in studies investigating the effects of temperature on embryonic development (Taylor et al. 2020; Tezak et al. 2018). Many studies report air or incubator temperatures to determine the effects of temperature on embryonic development, but future studies should ensure that they measure incubation temperature as close to the egg as possible.

Overall, the role of moisture in influencing non-squamate PSR is not clearly defined. Research has been biased toward investigations in the Testudines, with comparison among studies difficult due to differences in experimental conditions (e.g., egg arrangement, substrate type) and reported measurements of moisture. Further research is required to identify whether moisture can directly influence PSR and if so, to identify the mechanism. It is currently thought that the interaction between moisture and temperature has the largest effect on sex determination (Sifuentes-Romero et al. 2017a), highlighting the importance of considering multiple environmental variables when investigating the effects of incubation conditions on hatchling phenotypes. Investigations into the effect of moisture during incubation are also recommended for other reptile taxa (i.e. non-Testudines).

#### Body size

Increases in moisture during incubation generally result in the production of heavier and longer hatchlings in freshwater and sea turtles (Table 1). Some of this effect is likely explained by eggs in wet substrates absorbing more water than eggs in dry substrates (Tracy et al. 1978). Turtle eggs on wet substrates generally absorb water during the first half of incubation and then lose water as development continues. In comparison, eggs on dry substrates generally decrease in mass throughout incubation (Packard et al. 1982a). Thus, embryos that develop on wet substrates have greater access to water, are more hydrated and are, therefore, heavier than embryos that develop on dry substrates (Packard et al. 1988). Studies on the effects of moisture on crocodilian hatchlings are lacking, but as crocodilian eggshells are largely resistant to water uptake or loss, the response of embryos to moisture changes are likely to be limited (Ferguson 1981; Packard et al. 1982b).

Increased moisture levels in sea turtle nests during incubation results in hatchlings converting more yolk mass into body mass, thus hatching at a larger size (Hewavisenthi et al. 2001; Miller et al. 1987). Similarly, freshwater snapping turtle embryos incubated on wet substrates mobilised more protein and lipids from the yolk and had higher tissue hydration levels than embryos incubated on dry substrates, resulting in larger body size at hatching (Packard et al. 1988). However, the mechanisms behind this remain unknown. One possible explanation is that drier incubation conditions lead to higher blood viscosity in the developing embryo, reducing the rate at which nutrients can be converted into body tissues (Packard and Packard 1986, 1989). However, Bilinski et al. (2001) found that calcium mobilisation from eggshell to embryo in leatherback turtle embryos was higher in drier incubation conditions. Additionally, snapping turtle embryos incubated on wet (− 150 kPa) substrates consumed more oxygen and produced more carbon dioxide than embryos on dry (− 850 kPa) substrates (Miller and Packard 1992). The greater metabolic rate of embryos incubated on wet substrates was attributed to their greater mass, and embryos from wet substrates did not convert yolk more efficiently than embryos from dry substrates. Thus, substrate moisture concentration did not alter mass-specific metabolic rate and instead, resulted in embryos from wet substrates continuing along the same growth trajectory as embryos from dry substrates, but for a longer duration and to a larger body size at hatching (Miller and Packard 1992). McGehee (1990) found that carapace length in loggerhead turtle (*Caretta caretta*) hatchlings decreased with increasing moisture levels from 0% w/w water concentration to 24% w/w concentration. Sea turtle nests are typically in the 2–5% w/w range (Wood et al. 2000), so it is possible that the moisture levels used by McGehee (1990) were too high, resulting in reduced embryonic growth and smaller hatchling size. Indeed, very high moisture is often associated with reduced hatching success in loggerhead turtles (Foley et al. 2006). Incubation moisture levels do not generally influence post-hatching growth rates in testudines (Bobyn and Brooks 1994; Brooks et al. 1991; McKnight and Gutzke 1993). However, some studies have observed faster post-hatching growth rates in sea turtle hatchlings incubated in wetter conditions (Erb et al. 2018), suggesting that further studies are required.

Embryos are generally less sensitive to moisture than they are to temperature (Packard et al. 1989a), but in circumstances where moisture concentrations are very high or low, moisture can play a larger role than temperature in determining embryonic growth and survival (Cagle et al. 1993). Additionally, moisture may directly influence certain processes in developing embryos. For example, snapping turtles embryos incubated in wet conditions mobilised more lipid and protein from the yolk than embryos incubated in dry conditions, but temperature had no effect on lipid mobilisation (Packard et al. 1988). Higher moisture levels appear to produce larger and heavier hatchlings within a certain range, but extreme moisture levels outside this range can have negative effects on body size and growth. Low moisture levels potentially increase embryo blood viscosity to levels that limit the mobilisation of nutrients and oxygen and thus reduce hatchling body size (Packard 1991). However, future research should ensure that experimental moisture levels cover a wide enough range to capture potential responses (e.g., Rimkus et al. 2002), as only moisture levels above or below critical levels may impact tissue development via yolk mobilisation (Hewavisenthi et al. 2001) or blood viscosity (Packard 1991). For example, natural nests of painted turtle eggs experienced water potentials that ranged from − 0 to − 77 kPa, and within this range, the quantity of water exchanged between the eggs and external environment, did not influence hatchling dry or wet mass (Ratterman and Ackerman 1989). In contrast, Packard et al. (1981) incubated painted turtle eggs at − 130, − 375 and − 610 kPa and found that higher water potentials produced heavier hatchlings than low moisture levels. These two conflicting studies can be explained by the range of water potentials used for testing. Results from Ratterman and Ackerman (1989) may reflect the role of moisture in natural nests in non-drought years, while Packard et al. (1981) may reflect potential responses to reduced moisture during years of drought. Therefore, researchers should not only ensure that experimental treatments cover a wide enough range to capture potential responses, but also consider ecologically relevant treatments by measuring conditions in natural nests.

#### Locomotor performance

The majority of research on possible effects of moisture during incubation on locomotor performance has involved snapping turtles and sea turtles (Table 2). Turtle hatchlings incubated in wet conditions are generally faster swimmers and crawlers (Finkler 1999; Miller 1993; Miller et al. 1987) and also show a smaller reduction in crawling speed after desiccation compared to hatchlings incubated in dry conditions (Finkler 1999). There are few studies outside the Testudines.

There are several possible explanations for improved locomotor performance of some turtle hatchlings incubated in wet conditions. The first is that better performance is a result of the hatchling’s larger size (Miller 1993), although this is not always the case (Gatto and Reina 2020). Another possibility is that hatchlings incubated in wetter conditions accumulate lactate more slowly than hatchlings incubated on or within dry substrates (Miller et al. 1987) or may be more hydrated than hatchlings from dry nests (Gatto and Reina 2020). Hatchlings incubated in dry environments have larger residual yolk mass relative to their body mass (Packard et al. 1988), and may require increased anaerobic energy expenditure to carry this additional yolk mass that is not contributing to locomotion (Miller et al. 1987). However, hatchlings with larger yolk reserves will have access to greater energy reserves when moving this mass (Radder et al. 2004). Last, it is possible that moisture may directly or indirectly influence embryonic muscle development, but the mechanisms behind these potential effects are unknown and further investigation is required.

Hatchlings incubated in wetter conditions are generally stronger and faster than hatchlings incubated in dry conditions, although studies have been heavily biased toward the Testudines and further investigation is recommended for other reptile taxa. Hypotheses for direct and indirect moisture-dependent effects on locomotor performance require further testing.

#### Hatching success and developmental rate

Excess moisture or inundation during incubation can result in decreased hatching success or even loss of the entire clutch (Caut et al. 2010; Kofron 1989; Villamarín-Jurado and Suárez 2007). While non-squamate eggs can be quite resistant to brief or intermittent inundation from rainfall, river flooding or unusually high tides (Caut et al. 2010; Cedillo-Leal et al. 2017; Pike et al. 2015), repeated stress due to excessive moisture almost always leads to embryonic mortality (Foley et al. 2006). Hatching success after rainfall or flooding varies depending on the elevation of egg clutches within a landscape (Kraemer and Bell 1980; Kushlan and Jacobsen 1990) and the stage of embryonic development (Cedillo-Leal et al. 2017; Limpus et al. 2020; Rafferty et al. 2017). Inundation appears to limit oxygen supply to the developing embryos such that late stage embryos, with higher metabolic demands, are more sensitive to oxygen deprivation than early-stage embryos (Cedillo-Leal et al. 2017).

Hatching success varies significantly among taxa but is generally greatest at intermediate moisture levels (Foley et al. 2006; Packard et al. 1991). Species-specific differences in sensitivity to moisture concentrations likely reflect their adaptation to surrounding environmental conditions (Table 3). For example, desert tortoises (*Gopherus agassizii*) have maximum hatching success in drier substrates (Spotila et al. 1994), while painted turtles and snapping turtles experience highest hatching success in much wetter conditions (Packard et al. 1987, 1989b, 1991; Paukstis et al. 1984). Thus, each species’ hatching success is maximised in their respective dry or wet habitats. These contrasting responses to moisture during incubation may be attributable to differences in permeability between rigid and soft-shelled turtle eggs (Booth 2002; Packard et al. 1999).

Overall, eggs incubated in dry conditions generally hatch earlier than those in wet conditions (McGehee 1990; Miller 1993; Packard et al. 1981, 1985a, 1991) and moisture appears to affect hatching success, although the sensitivity of species to moisture concentrations varies (Rimkus et al. 2002; Thompson et al. 1996; Thompson 1990). Non-squamate embryos are generally resistant to intermittent periods of extreme high or low moisture, but extended or regular exposure to very wet or very dry conditions has been shown to reduce hatching success in crocodilians and testudines. Future research should investigate taxa-specific responses to moisture during incubation, noting that habitat preferences and egg types likely influence these responses.

### Salinity

Elevated salinity is becoming increasingly concerning in terrestrial, freshwater and marine ecosystems (IPCC 2014; Nielsen et al. 2003) because of sea level rise, anthropogenic activities such as mining and agriculture, and increased evaporation rates (Cañedo-Argüelles et al. 2013; Kaushal et al. 2018). Increases in salinity in the incubation environment usually decrease hatching success in turtles (Bower et al. 2013; Bustard and Greenham 1968; Foley et al. 2006) and crocodilians (Mazzotti 1989), although this is not always the case (Table 3; Bézy et al. 2015). Freshwater and sea turtle hatchlings tend to be smaller when incubated in substrates with higher salinities compared to less saline environments, displaying phenotypes that are similar to those seen at low water potentials, i.e., dry conditions (Table 1; Bézy et al. 2015; Bower et al. 2013). It is possible that regulating and removing excess salts requires considerable energy and reduces the energy available for growth (Holliday et al. 2009). Similarly, American crocodile (*Crocodylus acutus*) eggs sprayed with seawater had lower egg mass, while eggs sprayed with fresh water increased in mass (Bustard and Greenham 1968), perhaps indicating that increased salinity interferes with normal egg metabolism and/or osmotic gradients outside of the egg. Seawater has a water potential of approximately − 2000 kPa, though beach water potentials are generally closer to − 50 kPa (Ackerman et al. 1997). Thus, the presence of seawater in nests, which has a lower water potential than eggs, may draw water osmotically out of the egg.

High salinity generally appears to have similar effects as low moisture on non-squamate embryos. For example, high salinity during incubation is associated with low hatching success and decreased hatchling size. Furthermore, embryo and hatchling traits seem to be less sensitive to changes in salinity than changes in temperature. More research is needed to elucidate the effects and mechanisms of salinity on hatchling phenotypes among non-squamates.

## The importance of monitoring interactions among environmental factors

Studies investigating how hatchling phenotypes are impacted by incubation conditions typically manipulate or test a single environmental factor. However, the interconnectedness of weather variables means that change in a single factor without concomitant changes in one or more other factors is unlikely in the natural setting. Variation in even a single environmental factor will, therefore, likely result in multiple alterations to incubation conditions that may vary among individual clutches. However, presumably for simplicity and practicality, few studies investigate how simultaneous changes in multiple environmental factors may influence one another and subsequently affect hatchling phenotypes. Here, we discuss the need to consider multiple environmental variables and assert that this approach provides a more sophisticated understanding of how incubation conditions influence hatchling traits.

### How do environmental factors influence one another?

**Fig. 1:**
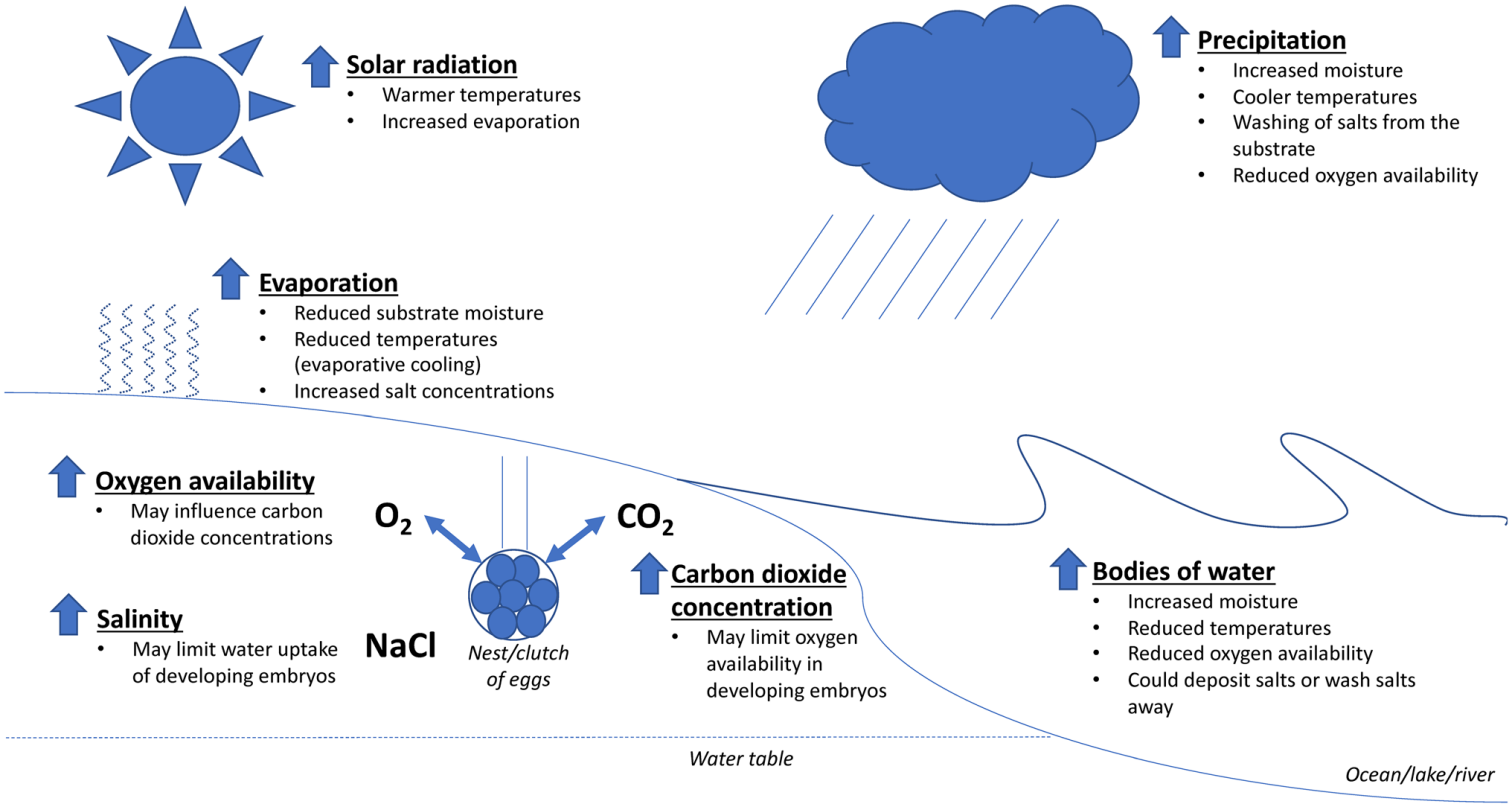
A diagrammatic representation of how environmental variables interact and influence nest conditions. Bodies of water represent both aboveground and underground water sources such as oceans, lakes, rivers and the water table, as well as areas such as valleys where water can collect and pool. The listed responses to bodies of water represents the likely changes to environmental variables as a nest becomes closer to that body of water

In broad terms, incubation conditions are largely driven by the regional and local climate (Packard et al. 1985b). However, finer scale variation in incubation conditions arises due to the presence and interaction of multiple environmental factors, such as temperature, moisture, gas concentrations, salinity and properties of the nest substrate. Temperature and moisture are the main determinants of incubation conditions within clutches of eggs (Table 4; Fig. 1), and this combination is accordingly the most studied. Both factors influence each other and also have measurable effects on oxygen concentration and salinity (Ackerman et al. 1997; Chen et al. 2010; Foley et al. 2006; Lutz and Dunbar-Cooper 1984). In comparison, the effect of oxygen concentration and salinity on moisture, temperature or each other is limited (Table 4: Fig. 1). However, experiments in non-squamate taxa have shown that both salt and oxygen concentrations can influence developing embryo’s responses to temperature and moisture (Bustard and Greenham 1968; Liang et al. 2015). Lastly, differences among substrate types and nest depth can alter the nest microclimate relative to the broader external environment and thus, alter the responses of developing embryos to the incubation environment (Booth 2006; Mitchell and Janzen 2019; Mortimer 1990; Seymour and Ackerman 1980). Thus, the nest environment is dynamic, complex and difficult to predict.

**Table 4:** The interacting effects of environmental variables within non-squamate nests

	Temperature	Moisture	Oxygen concentration	Salinity
Increased temperature		Increased evaporative rates resulting in reduced nest moisture levels^A^	Nest temperature generally increases during incubation due to metabolic heat production of the embryos. Both the increased temperatures and the increased development and size of the hatchlings results in increased oxygen demands for the embryos and results in decreased oxygen availability within the nest^B^ Temperature can also influence diffusion rates and gas densities within clutches^C^	Increased temperatures do not directly influence salt concentration within nests, but increased temperatures can increase evaporative rates resulting in increased salt concentration within nests^A^
Increased moisture	Decreased temperature either via direct cooling or increased evaporative cooling^A,E,F^		Water displaces air in-between substrate particles resulting in reduced oxygen availability within the nest^A,I,J^	Depends on the salinity of the water. Seawater can deposit salts while rainfall can rinse the nest thereby reducing salinity^A,K^
Increased oxygen concentration	Oxygen concentration does not directly influence nest temperatures, but higher oxygen levels can help embryos be more resistant to thermal stress compared to embryos developing in low oxygen environments^D,L^	Oxygen concentration does not directly influence nest moisture but caiman embryos that had access to oxygen via air bubbles trapped on their rough shell had increased resilience to inundation compared to embryos with smooth shells^G^		Oxygen concentration does not influence salt concentration
Increased salinity	Salinity does not influence nest temperatures	Salt concentrations can influence water gradients and potential within nests. However, the effects of salt on the movement of water within nests is minimal^A^	Salinity does not directly influence oxygen concentrations within nests. However, increased salinity can result in increased metabolic stress for developing embryos. This can impact embryonic metabolic rates and the availability of oxygen within the nest^H^	

For salinity and oxygen concentration, we also list how they can modulate the response of developing embryos to other environmental variables

^A^Ackerman et al. (1997)

^B^Chen et al. (2010)

^C^Ackerman (1980)

^D^Liang et al. (2015)

^E^Houghton et al. (2007)

^F^Tezak et al. (2018)

^G^Cedillo-Leal et al. (2017)

^H^Bustard and Greenham (1968)

^I^Caut et al. (2010)

^J^Kam (1994)

^K^Foley et al. (2006)

^L^Smith et al. (2015b)

Studies of single environmental variables are vital for understanding how specific factors influence hatchling phenotypes under controlled conditions. However, as attention shifts from controlled experiments to understanding incubation conditions in situ, more research is needed to identify the effects of interacting environmental factors. This not only includes understanding how environmental factors influence one another, but also investigating how changes in one factor can influence an embryo’s subsequent response to a different factor. Future studies should aim to measure, control and report temperature, moisture and oxygen concentrations throughout incubation, even in studies that investigate the effects of only a single variable. Studies that manipulate thermal conditions may incubate eggs at different moisture levels that in turn impact oxygen concentrations, resulting in altered phenotypic responses. Reporting this information will allow for easier identification of potential sources of phenotypic variation, will facilitate future meta-analyses and will improve current models of hatchling phenotypic variation, providing a clearer and more accurate understanding of which combinations of environmental variables maximise reproductive fitness in adults than is currently available.

## What are the implications of altered incubation conditions for reptile populations?

### How might climate change affect hatchling phenotypes?

Increased air temperatures under anthropogenic climate change are predicted to negatively impact embryonic development, hatchling growth and overall survival in most reptile species. Research is most extensive on the impact of increased air and incubation temperatures on sea turtles, that are predicted to become smaller, lighter and generally less capable of survival under anthropogenic climate change. Small hatchlings that emerge with large yolk reserves may have greater endurance than large hatchlings with small yolk reserves, but in the case of sea turtles, these modest increases in endurance will not be enough to overcome reduced swimming speeds in predator-dense coastal waters and an inability to escape wave zones or currents (Cavallo et al. 2015). Small sea turtle hatchlings are also more vulnerable to predation than large hatchlings (Gyuris 2000; Janzen et al. 2000). Despite the negative effects of warmer incubation temperatures during embryonic development on locomotor performance (Sim et al. 2015), warmer ambient air and water temperatures may actually boost hatchling sea turtle locomotor performance (Booth and Evans 2011), because ambient temperature also influences non-squamate locomotor performance (Booth and Evans 2011). However, changes to incubation temperatures are likely to have a greater effect on hatchling phenotypes than ambient temperatures post-hatching, because the ability of embryos to thermoregulate is limited (Cordero et al. 2018; Telemeco et al. 2016). Predictions of embryonic and hatchling responses to climate change in freshwater turtles, crocodilians and tuataras are less extensive and require further investigation.

Current predictions focus on the effects of altered incubation temperatures on hatchling phenotypes and largely ignore the effects of other environmental variables. Environmental factors such as moisture, oxygen and salt concentrations are likely to vary significantly under anthropogenic climate change. Variation in these factors could mitigate or exacerbate thermal effects, further complicating predictions of phenotypic responses. For example, small increases to moisture caused by increased rainfall may have positive effects for hatching success in some non-squamate species by directly reducing incubation temperatures and by increasing evaporative cooling (Charruau 2012; Houghton et al. 2007; Laloë et al. 2020; Staines et al. 2020). Conversely, a decrease in rainfall may further exacerbate the effects of increased temperatures on hatching success. Expected increases in storm intensity (IPCC 2014), including extended deluges, are likely to decrease non-squamate hatching success because of flooding and submersion of eggs (Kam 1994). Thus, models of future responses to climatic variation that only consider thermal variation may be over or underestimating potential responses. Models of future climatic conditions and hatchling responses are complex and full of uncertainty, failing to consider multiple, influential environmental variables such as moisture, further limits their accuracy and usefulness.

Additional complications arise from physiological differences among species such as the permeability of the eggshell (Packard et al. 1982b), the physiological processes occurring inside the egg (Ackerman 1991), the ability of species to alter where they lay their eggs (Kamel and Mrosovsky 2004) and nesting phenology (Neeman et al. 2015). Altered environmental conditions can also influence maternal nutrition, body condition and thermoregulation, resulting in altered allocation of resources to embryos and altered nesting behaviour (Ma et al. 2014; Telemeco et al. 2010; Warner 2014; Price et al. 2004). Many studies have compared the relative importance of maternal investment and incubation conditions on hatchling phenotypes and reproductive success at the clutch and population levels (Gatto et al. 2020; Leivesley and Rollinson 2021; Roosenburg and Kelley 1996; Tezak et al. 2020; Wallace et al. 2007). However, the relative influence varies between species and even populations. Further research is required to identify the relative influence that altered environmental conditions have on offspring phenotypes directly during incubation and indirectly by altering maternal investment to reproduction.

These physiological and behavioural differences alter how hatchling phenotypes respond to climatic variation and must be considered when predicting responses to anthropogenic climate change. Key first steps include expanding the number of studies that examine the effects of multiple, interacting environmental variables on hatchling phenotypes and expanding models of future incubation conditions to include interacting environmental variables.

### What are the consequences for population viability?

It is critical to understand and predict how hatchling phenotypes respond to climatic variation to model the viability of populations under anthropogenic climate change and subsequently, manage and conserve these populations. The responses of populations to altered incubation conditions and hatchling phenotypes will vary but research has identified a number of important factors that determine the sensitivity of species and populations to anthropogenic climate change. Studies on crocodilians and tuataras are sparse, so here, we mainly use studies on the Testudines to identify potential population responses to altered incubation conditions and hatchling phenotypes.

Studies on the effects of climate-mediated changes in incubation conditions have generally focused on PSR and their long-term consequences for adult populations (Fuentes et al. 2010; Hays et al. 2017; Telemeco et al. 2013). Projected long-term increases in global temperatures (Hoegh-Guldberg et al. 2018; IPCC 2014) may result in increased production of one sex (i.e. males for FM species or females for MF and FMF species), resulting in unbalanced adult sex ratios and the risk of eventual population collapse in certain species (Hays et al. 2017; Santidrián Tomillo et al. 2014; Telemeco et al. 2013). However, some species, like sea turtles, may be resilient to biased primary and adult sex ratios, subject to a growth trade-off (i.e. feminisation increasing population growth rates until collapsing due to a lack of males) (Hays et al. 2017; Laloë et al. 2017; Santidrián Tomillo et al. 2021; Schwanz and Georges 2021). For instance, in sea turtles, differences in breeding periodicity between the sexes can balance operational sex ratios despite biased adult sex ratios (Hays et al. 2010, 2014; Santidrián Tomillo and Spotila 2020). Despite the importance of sex ratios, reductions in hatching success may have a larger effect on population viability. Embryonic mortality appears likely to impact population viability in Chelonians, potentially even before incubation conditions become extreme enough to substantially alter adult sex ratios (Hays et al. 2017; Laloë et al. 2017; Santidrián Tomillo et al. 2014, 2012).

**Fig. 2:**
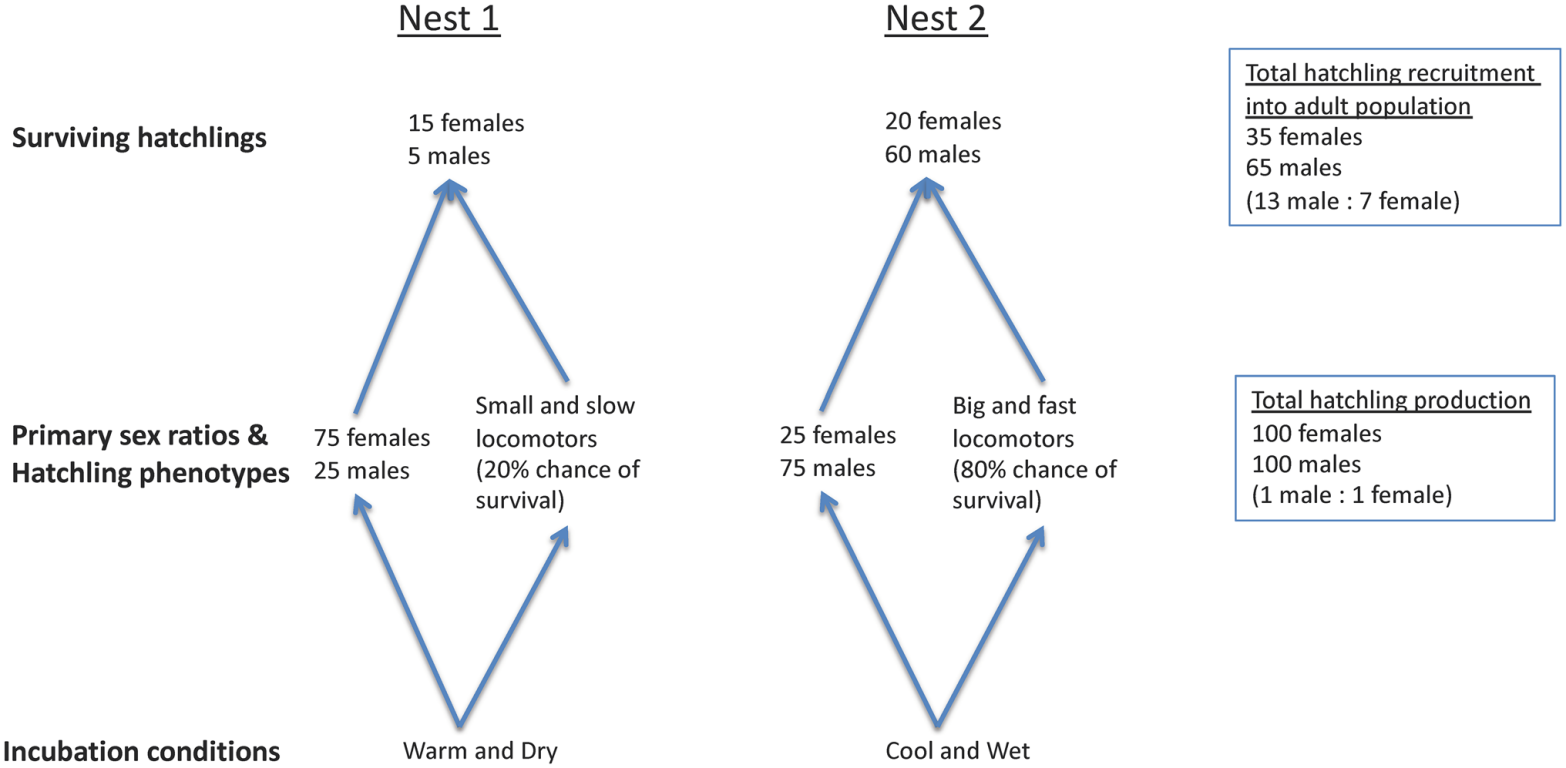
Co-variation in primary sex ratios and hatchling phenotypes with incubation conditions results in ‘filtered’ primary sex ratios. The sex ratios of hatchlings recruited into adult populations are altered from primary sex ratios, because the conditions that produce more hatchlings of a particular sex, in this case males, also produce bigger hatchlings that are faster runners/crawlers and are likely to have lower mortality rates (Santidrián Tomillo et al. 2014)

Furthermore, sex-specific differences in survival rates can significantly alter the sex ratios of hatchlings recruited into adult turtle populations (Girondot and Pieau 1993; Schwanz and Georges 2021; Steen et al. 2006). Generally, males and females from the same clutch do not differ in their locomotor performance or morphology (Booth et al. 2004; Marcó et al. 2010). However, variation in hatchling traits between clutches can alter hatchling recruitment in a sex-specific manner (Fig. 2). For example, cool and wet incubation conditions may result in a male-biased clutch of larger and faster hatchlings, while warm and dry incubation conditions may result in a female-biased clutch of smaller and slower hatchlings (Rivas et al. 2019). The larger and faster male-biased clutch may be more capable of chasing prey and escaping predators, and thus more likely to experience greater survival rates than the female-biased clutch (Gyuris 2000; Santidrián Tomillo et al. 2014). Thus, in this scenario, more males are likely to survive and be recruited into the adult population, even if the PSR of the two nests combined was approximately 1:1. It is possible that sex-specific survival rates (and thus sex ratios) may vary among life stages, but more cross-taxa research is needed to confirm this.

Nesting adults may be able to mitigate the effects of altered incubation conditions by adjusting their nesting behaviour, although their capacity to adjust is currently uncertain (Urban et al. 2014). Research has focused on nesting phenology and non-squamates generally start nesting earlier in response to increased temperatures (Cherkiss et al. 2020; Janzen et al. 2018; Lamont and Fujisaki 2014; Monsinjon et al. 2019; Schwanz and Janzen 2008; Weishampel et al. 2004, 2010) preceding the nesting season (Janzen et al. 2018; Lovich et al. 2012). Nesting females may also be able to select nest sites or depths that mitigate the effects of altered incubation conditions, if they are available (Czaja et al. 2020; Liles et al. 2019; Mitchell et al. 2008; Reboul et al. 2021; Refsnider and Janzen 2012; Staines et al. 2019).

It is important to note that climate effects on phenotypes are likely to be non-uniform and may even benefit certain taxa. For instance, sea turtle populations at higher latitudes may produce more balanced sex ratios and greater reproductive output under climate change (Montero et al. 2019). Gravid females may gain a reproductive benefit by laying their eggs during periods of the breeding season that produce higher quality hatchlings, or hatchlings of the less-common sex (Kamel and Mrosovsky 2005). Individuals or sub-populations that produce hatchlings of the less-common sex will become more valuable for maintaining population viability (Baptistotte et al. 1999; Stubbs et al. 2014) because of their ability to balance sex ratios at the population level (Bowen et al. 2005). Research to identify these valuable populations and maximise the production of the less-common sex should be prioritised.

Predictions of the consequences of altered incubation conditions for reptile populations must also consider whether incubation effects are long or short term. Current studies generally measure incubation effects over time periods of days or weeks, and few studies consider them over months or years (Refsnider et al. 2019). However, it is likely that the environment experienced by hatchlings post-emergence eventually overrides the effects of incubation conditions. Thus, studies that predict the effects of incubation environments on individual survival and on populations, should consider for how long incubation environment remain the primary determinant of hatchling traits before post-emergence influences become more important.

In summary, altered incubation conditions due to climate change may influence adult populations in four main ways: (1) altering PSR, (2) altering incubation conditions to influence hatchling phenotypes, survival and recruitment rates, (3) by giving hatchlings incubated under certain conditions long-term fitness advantages (including sex-specific survival rates) over other hatchlings, and (4) conferring reproductive advantages for females that nest in certain locations or at times that maximise hatchling quality and quantity. The degree of these changes is likely to vary due to the predicted heterogeneity of climate change and the capacity of individuals and populations to respond within necessary timeframes. Research on the effects of altered incubation conditions and hatchling phenotypes on adult populations is largely constrained to the Testudines and additional focus should be paid to crocodilian and tuatara populations.

## Future research directions

Published studies on the effects of incubation conditions on hatchlings of non-squamate reptiles have largely focused on temperature and have paid less attention to the effects of moisture, oxygen, substrate and other environmental factors such as salinity and oxygen concentration (Warner et al. 2018). Environmental variables other than temperature require more attention and in particular, the synergistic effects of multiple environmental variables need to be investigated in more detail to allow more accurate predictions of how non-squamate traits will be affected by future environmental variation.

Predicting possible responses to environmental change is also complicated by variation in the responses of non-squamate species, populations and clutches to altered incubation conditions. Identifying trends and exceptions among species and populations will be aided by considering the synergistic effects of interacting environmental factors and by standardising techniques used to measure hatchlings traits and environmental fluctuations. For example, recent studies report incubation moisture levels in terms of water content (%), either percentage volume or mass, and others report water potential (kPa). Future studies should report water potential wherever possible, as it drives water exchange across the eggshell and is more easily comparable among studies. Additionally, there can be up to 2 °C difference in the temperature measured at the incubator level and the egg level in the same study (Taylor et al. 2020; Tezak et al. 2018), so techniques for measuring thermal stress and heat tolerance in embryos also require standardisation (Hall and Sun 2020). Creating standard techniques will thereby facilitate the comparison of studies among species and populations.

A key tool for future research will be meta-analyses that examine how multiple phenotypic traits respond to environmental variation, e.g., Noble et al. (2018b). Currently, meta-analyses on the effects of environmental variables other than temperature on hatchling phenotypes are limited by a lack of studies generally and the focus of these studies on particular taxa e.g., sea turtles. However, when possible, future meta-analyses should incorporate multiple environmental factors, including temperature, to identify overall interacting effects on multiple traits. The creation of large, shared datasets (e.g., Noble et al. 2018a) will help identify potential avenues for further research and overall responses to incubation conditions. These analyses should also consider phylogenetic differences among species. Even if the mechanisms behind phenotypic plasticity in hatchling traits are conserved, there is considerable variation in the responses to incubation environment among non-squamate taxa. Thus, studies should pay particular attention to which taxa and species respond similarly or diverge in their response to incubation conditions.

At the individual level, recent research has begun to identify how incubation conditions alter embryonic development and hatchling traits, but the mechanisms behind the effects of incubation conditions should remain a research priority (Gangloff and Telemeco 2018; Taylor et al. 2020; Warner et al. 2018). Understanding how changes in the incubation environment are sensed by cells and how they influence gene expression and each individual’s physiology will help identify how non-squamates respond to environmental variation. Particularly, research on the genetic basis of developmental plasticity will help determine whether non-squamate populations will be able to adapt to climate change.

It is also important that future studies consider the potential impacts of altered incubation environments on population dynamics and viability. A key first step is identifying how long incubation conditions play a primary role in determining hatchlings traits and when, if at all, other factors such as food availability or thermal regimes, begin to override the effects of incubation conditions (Mitchell et al. 2018). A recent meta-analysis reported that the effects of incubation conditions can persist for at least a year post-hatching, although studies that reported the effects of incubation conditions at 365 days of age were few (*n* = 8) compared to studies that reported the effects of incubation conditions within 10 days of hatching (*n* = 140) (Noble et al. 2018b). Measuring the effects of incubation conditions over longer time periods and in natural systems will provide further information on the ecological relevance of incubation effects and the consequences for population distributions, dynamics and viability. Studies that consider the effect of incubation conditions on adult populations should also incorporate the potential responses of nesting females. Adult populations may shift the timing and location of nesting in response to altered environmental conditions (Cherkiss et al. 2020; Monsinjon et al. 2019; Weishampel et al. 2010). As a result of behavioural plasticity the actual conditions experienced by developing embryos may not change at the same rate as would be expected from ambient conditions alone.

Overall, temperature has been the focus of the majority of studies, but more research is required on other environmental factors and particularly, on the synergistic effects of multiple environmental factors. Combined with improving our understanding of the mechanisms that control genetic and phenotypic expression, and improving our understanding of the long-term effects of hatchling responses to altered incubation conditions, this will allow a greater understanding of the impacts on populations. Research has been most extensive in the Testudines, with the Cheloniidae (sea turtles), Chelydridae (snapping turtles) and Emydidae (terrapins and sliders) receiving the most attention within the order. The Testudinidae (tortoises) should receive more attention, particularly as a terrestrial comparison to aquatic species. Crocodilians have received less attention than Testudines and the majority of research has been conducted in North and Central America. Future research should expand the number of studies on crocodilians, focussing on expanding the number of species and populations.

## Conclusions

1. Research on the effects of incubation conditions on hatchling phenotypes in oviparous non-squamates has largely focused on the role of temperature. The impacts of other environmental factors such as moisture, oxygen concentration and salinity have been under-investigated, although it is clear that these factors may have significant biological impacts on non-squamate embryonic development.
2. Specifically, the current focus on temperature does not account for variation in other environmental factors (e.g., moisture) or the combined effects of multiple, interacting factors on hatchling phenotypes. As a result, most current predictions of non-squamate phenotypic responses to environmental fluctuations do not account for the full spectrum of changes that might be expected in response to climate change. In particular, crocodilians have received little attention compared to the Testudines. Additionally, tuataras also require further attention because of their unique physiology and evolutionary history as well as their southerly habitat and subsequent adaptation to low temperatures relative to other non-squamates. Future studies should also focus on species from Asia, South America and Africa rather than the well-studied continents of North and Central America, Europe and Australia.
3. Environmental variation is spatially and temporally heterogenous, resulting in a variety of species-specific responses among non-squamates. Moisture concentrations during development play an important role in sex determination, both indirectly by modulating the effects of temperature on developing embryos and possibly directly via unknown mechanisms. Elevated moisture concentrations generally result in the production of larger hatchlings, while intermediate concentrations result in the highest hatching success and locomotor performance. High moisture concentrations can limit oxygen availability to embryos and limit the removal of carbon dioxide, resulting in smaller hatchlings and reduced hatching success. Hypercapnia may result in the production of more females in Testudines. High salt concentrations generally produce hatchlings with similar phenotypes to those that developed in low moisture environments.
4. Based on the information available regarding climatic warming and predicted responses, expected changes to PSR will eventually lead to population-wide sex ratio imbalances, while changes to hatchling morphology and locomotor performance will impact hatchling recruitment, possibly in a sex-dependent manner. Predicted increases in embryonic and hatchling mortality may have a greater impact on non-squamate adult populations than altered PSR but identifying the consequences of altered incubation conditions for adult populations is difficult. Increases in rainfall and sea level rise have the potential to offset the effects of warmer nesting sites and produce higher quality offspring, and, for species with TSD, hatchlings of the less-common sex. However, research on the relative effects of PSR and embryonic mortality on population viability has focused on the Testudines.
5. Understanding phenotypic responses to dynamic, multifaceted nesting environments is vital for conserving and managing oviparous species. To predict the impact that environmental variation will have on embryonic development, it is necessary to understand how interacting environmental factors may alter hatchling phenotypes and to incorporate this knowledge into population models.
6. Future research should further investigate phenotypic responses to multiple environmental variables in both field and laboratory studies. Additionally, studies have not thoroughly examined the role of local substrate characteristics in influencing incubation conditions, so research is needed to examine these characteristics to determine how current nesting habitat may change under predicted climatic variation. Finally, research should continue to investigate how incubation conditions ultimately shape adult populations, as well as how adults may alter their behaviour to optimise incubation conditions for their offspring.
7. Non-squamates are a diverse and ecologically important group of vertebrates that are particularly valuable as model species for studies on the effects of environmental variation during development. However, their diversity, both in the squamates and non-squamates makes generalising among them difficult and highlights the importance of analysing patterns within a phylogenetic framework and strategically directed research.

## Supplementary Information

Below is the link to the electronic supplementary material.

Supplementary file1 (DOCX 50 KB)

## Data Availability

N/A.

## Code availability

N/A.
