# Supplementary material for: A review of the effects of incubation conditions on hatchling phenotypes in non-squamate reptiles
Source: J Comp Physiol B. 2022 Feb 10;192(2):207–33. doi: 10.1007/s00360-021-01415-4 (PMC8894305; doi:10.1007/s00360-021-01415-4)
Supplement: Supplementary file 1 — Supplementary file1 (DOCX 50 KB) [file 360_2021_1415_MOESM1_ESM.docx]

Table S1: The temperature-dependent sex determination (TSD) patterns and pivotal temperatures of various oviparous non-squamate orders. For species without a specific pivotal temperature, the best approximation (range of temperatures) are given.

| **Order** | **Family** | **Species** | **TSD Pattern** | **Pivotal temperature/s (°C)** | **Reference** |
| --- | --- | --- | --- | --- | --- |
| Testudines | Chelonidae | *Eretmochelys imbricata* | MF | 29.2 | Dobbs et al. (2010) |
|  |  | *Eretmochelys imbricata* | MF | 29.2-29.6 | Wibbels (2003) |
|  |  | *Chelonia mydas* | MF | 29.2-29.3 | Godfrey and Mrosovsky (2006) |
|  |  | *Chelonia mydas* | MF | 28.8-30.3 | Wibbels (2003) |
|  |  | *Chelonia mydas* | MF | ~29 | King et al. (2013) |
|  |  | *Caretta caretta* | MF | Mon Repos- 27.7  Heron Island- 28.7 | Limpus et al. (1985) |
|  |  | *Caretta caretta* | MF | 28.7-30 | Wibbels (2003) |
|  |  | *Lepidochelys olivacea* | MF | 30-31 | Wibbels (2003) |
|  |  | *Lepidochelys kempii* | MF | 30.2 | Wibbels (2003) |
|  |  | *Natator depressus* | MF | 29.4 | Stubbs et al. (2014) |
|  | Dermochelyidae | *Dermochelys coriacea* | MF | 29.4-29.5 | Wibbels (2003) |
|  | Chelydridae | *Chelydra serpentina* | FMF | 20.3-24.2 & 25.6-28.2 | Ewert et al. (2005) |
|  | Emydidae | *Malaclemys terrapin* | MF | 28.29 | Burke and Calichio (2014) |
|  | Geoemydidae | *Mauremys japonica* | MF | 28.8 | Okada et al. (2010) |
|  |  |  |  |  |  |
| Crocodilia | Crocodylidae | *Crocodylus acutus* | FMF | 31.1 & 33.6 | Charruau et al. (2017) |
|  |  | *Crocodylus acutus* | FMF | 31 & 32.5 | Charruau (2012) |
|  |  | *Crocodylus johnstoni* | FMF | 31.5 & 32.5 | Lang and Andrews (1994) |
|  |  | *Caiman crocodilus* | FMF | 31.5 & 34 | Lang and Andrews (1994) |
|  |  | *Caiman latirostris* | FMF | 32-33 & 34-34.5 | Marcó et al. (2017) |
|  | Alligatoridae | *Alligator mississipiensis* | FMF | 31.8 & 33.8 | Lang and Andrews (1994) |
|  |  |  |  |  |  |
| Rhynchocephalia | Sphenodontia | *Sphenodon guntheri* | FM | 22 | Mitchell et al. (2006) |

Table S2: The effect of incubation temperature on mass, morphology and post-hatching growth rates. Studies are allocated based on the conditions that produced the largest hatchlings and fastest growth rates.

|  | **With warmer temperatures** | **With cooler temperatures** | **With intermediate temperature** | **No effect of temperature** |
| --- | --- | --- | --- | --- |
|  | **Increased mass** | | | |
| Turtle | de Souza and Vogt (1994) | Gutzke and Packard (1987) | Fisher et al. (2014); Hewavisenthi et al. (2001) | Booth and Evans (2011); Fisher et al. (2014); Ischer et al. (2009); Janzen and Morjan (2002); Reece et al. (2002); Wood et al. (2014) |
| Tortoise |  | Spotila et al. (1994) |  |  |
| Crocodile |  |  | Marcó et al. (2010) | Allsteadt and Lang (1995); Hutton (1987); Webb and Cooper-Preston (1989) |
|  | | | | |
|  | **Increased carapace length/SVL** | | | |
| Turtle |  | Booth and Evans (2011); Gutzke and Packard (1987); Maulany et al. (2012); Micheli‐Campbell et al. (2011); Reece et al. (2002); Sim et al. (2015) | Fisher et al. (2014); Hewavisenthi et al. (2001) | Ashmore and Janzen (2003); Booth and Astill (2001) |
| Crocodile |  | Hutton (1987) | Allsteadt and Lang (1995), (Marcó et al. 2010) | Joanen et al. (1987); Webb and Cooper-Preston (1989) |
|  | | | | |
|  | **Increased carapace width** | | | |
| Turtle |  | Booth and Evans (2011) | Fisher et al. (2014); Hewavisenthi et al. (2001) | Booth and Astill (2001) |
|  | | | | |
|  | **Increased growth rates (post-hatching)** | | | |
| Turtle | Booth et al. (2004); Janzen and Morjan (2002); Roosenburg and Kelley (1996) | Brooks et al. (1991); Rhen and Lang (1995) | McKnight and Gutzke (1993) | Steyermark and Spotila (2001) |
| Tortoise |  |  | Spotila et al. (1994) |  |
| Crocodile | Hutton (1987) |  | Joanen et al. (1987) |  |

Table S3: The response of various measures of locomotor performance to different incubation temperatures. The temperature at which each trait is highest is identified and temperatures where no difference in that trait was observed are separated by ‘&’. For studies that analysed incubation temperatures as a continuous variable, we report the range of temperatures observed and where the trait was highest, if it was highest at an intermediate temperature.

| **Locomotor trait** | **Response to incubation temperature** | **Incubation temperatures** | **Species** | **Reference** |
| --- | --- | --- | --- | --- |
| Power stroke rate | Slower at cooler temperatures | 26 < 28 & 30 | *Chelonia mydas* | Booth et al. (2004) |
|  |  | 26 < 28 & 30 (2000)  25.5 < 30 (2002) | *Chelonia mydas* | Burgess et al. (2006) |
|  |  | Ranged from 28.5 to 32.4 ^A^ | *Chelonia mydas* | Ischer et al. (2009) |
| Time spent power stroking | Less time at cooler temperatures | 25.5 < 30 | *Chelonia mydas* | Burgess et al. (2006) |
| Force produced per power stroke | More force at cooler temperatures | Warm- 30.7  Cool- 29.1 ^B^ | *Chelonia mydas* | Booth and Evans (2011) |
|  |  | Ranged from 27.9-30.9 (2010 & 2011)  31-32.6 (2012) ^A^ | *Caretta caretta* | Sim et al. (2015) |
| Crawling/running speed | Faster at warmer temperatures | Ranged from 28.5 - 32.4 | *Chelonia mydas* | Ischer et al. (2009) |
|  |  | Ranged from 28.1 – 32.7 | *Caretta caretta* | Read et al. (2013) |
|  |  | Ranged from 29.6 – 32.2 | *Caretta caretta* | Wood et al. (2014) |
|  | Faster at intermediate temperatures | Ranged from 27 -31 but highest at 29-30 | *Caretta caretta* | Fisher et al. (2014) |

^A^ Incubation occurred in relocated nests on the nesting beach

^B^ Incubation occurred in relocated nests on the nesting beach. Nests were allocated to warm or cool treatment groups with the mean temperature of those groups provided.

^C^ Incubation occurred at fluctuating temperatures.

Table S4: Minimum and maximum hatching success in various non-squamate taxa and the temperatures that produced those results.

| **Order** | **Family** | **Species** | **Maximum hatching success** | **Temperature** | **Minimum hatching success** | **Temperature** | **Reference** |
| --- | --- | --- | --- | --- | --- | --- | --- |
| Crocodilia | Alligatoridae | *Alligator mississipiensis* | 83% | 32.8°C | 76.20% | 30.6°C | Joanen et al, 1987 |
|  |  | *Caiman latirostris* | 65% | 31°C | 16.20% | 34.5°C | Piña et al, 2003 |
|  | Crocodylidae | *Crocodylus niloticus* | 83% | 31°C | 69.00% | 34°C | Hutton, 1987 |
|  |  | *Crocodylus porosus* | ~73% | 31°C | ~25% | 36°C | (Webb and Cooper-Preston 1989) |
|  |  | *Crocodylus johnstoni* | 63% | 30°C | 0% | 26°C | (Webb et al. 1983) |
|  |  |  |  |  |  |  |  |
| Rhynchocephalia | Sphenodontidae | *Sphenodon punctatus* | 100% | 21°C | 87.50% | 18°C | Nelson et al, 2004 |
|  |  | *Sphenodon punctatus* | 62% | 20°C | 0% | 15°C | Thompson (1990) ^A^ |
|  |  |  |  |  |  |  |  |
| Testudines | Chelidae | *Elusor macrurus* | 89% | 26°C & 29°C | 56.00% | 32°C | Micheli-Campbell et al, 2011 |
|  | Cheloniidae | *Caretta caretta* | 69.20% | 29°C | 33.30% | 32°C | Fisher et al, 2014 |
|  |  | *Chelonia mydas* | 75% | 28°C | 70% | 30°C | Booth et al, 2004 |
|  |  | *Chelonia mydas* | 80% | 30°C | 75.00% | 26°C | Burgess et al, 2006 ^G^ |
|  |  | *Chelonia mydas* | 87% | 25.5°C | 58.30% | 30°C | Burgess et al, 2006 ^F, H^ |
|  |  | *Chelonia mydas* | 71% | 27.6°C | 40.00% | 30°C | Godfrey and Mrosovsky, 2006 |
|  |  | *Chelonia mydas* | 100% | 29°C | 15% | 33°C | Miller and Limpus (1981) |
|  |  | *Eretmochelys imbricata* | 80-100% | 28°C & 29.5°C | 40-80% | 32.5°C | Dobbs et al, 2010 |
|  | Emydidae | *Chrysemys picta* | 83% | 26°C | 77.00% | 30°C | Janzen and Morjan, 2002 ^I^ |
|  |  | *Emydoidea blandingii* | 95% | 26.5°C | 0.00% | 22°C | Gutzke and Packard, 1987b |
|  | Testudinoidea | *Gopherus agassizii* | 96% | 28.1°C | 29.00% | 35.3°C | Spotila et al, 1994 ^J^ |
|  | Trionychidae | *Pelodiscus sinensis* | 97% | 27°C | 44.00% | 23°C | Du and Ji, 2003 |
|  |  | *Pelodiscus sinensis* | 96.60% | 28°C | 68% | 34°C | Ji et al, 2003 |
|  |  | *Trionyx triunguis* | 88.3% | 30°C | 0% | 24°C | Leshem et al. (1991) |

^A^ Incubation treatments included moisture treatments. Calculation of hatching success for each temperature was the average of the moisture treatments at that temperature.

^B^ Incubation temperatures were changed midway through incubation. Treatment groups were combined for analysis (25/25, 25/28 & 28/28 vs. 28/30 & 30/30).

^C^ Only two incubation temperatures (25°C and 29°C.)

^D^ Each temperature split into dry (-220kPa) and wet (0kPa) moisture treatments.

^E^ Mortality was very low (8.4%) in all treatments

^F^ Only two incubation temperatures- 25°C and 30°C

^G^ 2000 experiments

^H^ 2002 experiments

^I^ Only two incubation temperatures: 26°C and 30°C

^J^ Only 0.4% moisture treatments included here
